# Liquid Biopsy in Non-Small Cell Lung Cancer

**DOI:** 10.3389/fmed.2016.00069

**Published:** 2016-12-23

**Authors:** Miguel A. Molina-Vila, Clara Mayo-de-las-Casas, Ana Giménez-Capitán, Núria Jordana-Ariza, Mónica Garzón, Ariadna Balada, Sergi Villatoro, Cristina Teixidó, Beatriz García-Peláez, Cristina Aguado, María José Catalán, Raquel Campos, Ana Pérez-Rosado, Jordi Bertran-Alamillo, Alejandro Martínez-Bueno, María-de-los-Llanos Gil, María González-Cao, Xavier González, Daniela Morales-Espinosa, Santiago Viteri, Niki Karachaliou, Rafael Rosell

**Affiliations:** ^1^Laboratory of Oncology, Pangaea Biotech, Quirón Dexeus University Hospital, Barcelona, Spain; ^2^Instituto Oncológico Dr Rosell, Quirón Dexeus University Hospital, Barcelona, Spain; ^3^Cancer Biology and Precision Medicine Program, Catalan Institute of Oncology, Germans Trias i Pujol Health Sciences Institute and Hospital, Badalona, Spain

**Keywords:** ctDNA, ctRNA, CTCs, exosomes, tumor-educated platelets, mutations, gene fusions, lung cancer

## Abstract

Liquid biopsy analyses are already incorporated in the routine clinical practice in many hospitals and oncology departments worldwide, improving the selection of treatments and monitoring of lung cancer patients. Although they have not yet reached its full potential, liquid biopsy-based tests will soon be as widespread as “standard” biopsies and imaging techniques, offering invaluable diagnostic, prognostic, and predictive information. This review summarizes the techniques available for the isolation and analysis of circulating free DNA and RNA, exosomes, tumor-educated platelets, and circulating tumor cells from the blood of cancer patients, presents the methodological challenges associated with each of these materials, and discusses the clinical applications of liquid biopsy testing in lung cancer.

## Introduction

The so-called “liquid biopsy” is quickly moving from research into clinical practice in lung cancer, as well as in other human malignancies. Although its full potential has not yet been reached, the “liquid biopsy” is no longer a promise but a reality that is allowing a better treatment selection and monitoring of lung cancer patients in hospitals and oncology departments worldwide. We can already foresee a day when “liquid biopsy”-based tests will be as widespread and useful as “standard” biopsies and imaging techniques, offering invaluable diagnostic, prognostic, predictive, and monitoring information. In this mini review, we will summarize the state of the art in this exciting area, placing a particular emphasis on the clinical utility of the “liquid biopsy” and the variety of applications, methodologies, and results that can be derived from it.

“Liquid biopsies” are usually defined as tests done in blood samples or other body fluids. In the case of cancer patients, the objective of those tests is to detect materials originated in the tumor. Although the term “liquid biopsy” is universally used, many pathologists argue that it is incorrect. The so-called “liquid biopsies,” they claim, are not true biopsies. A “true” biopsy is usually performed by a surgeon or a pneumologist and involves the extraction of sample cells or tissues that are subsequently examined by a pathologist under a microscope, commonly after some kind of fixation and staining. Paraffin embedding is also widespread. In contrast, “liquid biopsies” are not obtained by surgeons; involve the extraction of blood or other fluids and not of solid tissues, pathologists only occasionally intervene and fixation, embedding, or staining are equally infrequent. In addition to the “biopsy” half, the “liquid” half in the term “liquid biopsy” can also be misleading. The materials originated in the tumor that are to be detected in such “biopsies” are never liquid. Some of them are cells or fragments of cells, such as circulating tumor cells (CTCs), exosomes, or tumor-educated platelets (TEPs); others are nucleic acids dissolved in the blood, such as circulating tumor DNA or RNA (ctDNA, ctRNA). Each of these materials offers unique opportunities to test different biomarkers and analyze particular characteristics of the tumors (Table 1).

**Table 1:** **Biological materials that can be isolated from liquid biopsies and their applications in lung cancer**.

Material	Applications
Circulating tumor DNA (ctDNA)	Somatic mutations^a^
	DNA methylation changes
	Copy number alterations
ctRNA	Gene fusion
	Splicing variants
Tumor-educated platelets	Gene fusions
	Splicing variants
	Cancer diagnosis
	RNA profiling
Exosomes	Gene fusions
	Splicing variants
	miRNA analyses
	RNA and protein-based molecular profiling
Circulating-tumor cells (CTCs)	Monitoring (total CTC counts)^b^
	Culture of CTCs
	DNA, RNA, and protein-based molecular profiling
	Somatic mutations
	Gene fusions

*Applications used in routine clinical practice in (^a^) NSCLC or (^b^) metastatic breast, prostate, and colon cancer patients. Unmarked, research use*.

The differences between a “real” and a “liquid” biopsy—or “liquid sample,” as the pathologists would probably prefer to call them—explain the advantages of the latter. “Liquid” biopsies will never replace real biopsies, which are irreplaceable sources of information that cannot be obtained by any other means, such as tumor type and histology. However, they offer all sorts of additional data that cannot be obtained in any other way. In patients who cannot be biopsied, or where biopsies do not have enough tissue, “liquid biopsy” is the only alternative to perform genetic testing for targeted therapy. Also, in patients with advanced disease, it is not feasible to obtain biopsies of every metastasic site. But blood reaches both the primary tumor and the metastases, and materials coming from all can be found in a “liquid biopsy.” Finally, unlike “real” biopsies, blood can be repeatedly obtained without the risk of comorbidities and used to monitor the course of the disease, including early detection of response and relapse or emergence of resistance to a particular therapy.

## Circulating Tumor DNA

Circulating free DNA (cfDNA) can be found dissolved in plasma and serum, at variable amounts. In the case of cancer patients, a fraction of the cfDNA is tumor derived, and ctDNA represents from less than 0.1% to more than 10% of the total cfDNA. This percentage has been shown to depend on stage, tumor burden, vascularization of the tumor, biological features like apoptotic rate and metastatic potential of the cancer cells, and factors affecting the blood volume of the patient (1, 2). In addition, variations on the relative abundance of ctDNA correlate with response to therapy (3–5). ctDNA is released by passive mechanisms, such as lysis of apoptotic and necrotic cells or digestion of tumor cells by macrophages, and also by active mechanisms. In this respect, cfDNA shows and enrichment in 150–180 bp fragments typical of the nucleosomal pattern of DNA fragmentation during apoptosis (6–9). The ctDNA carries the same somatic alterations as the tumor itself and can be used to detect clinically relevant mutations such as those in the epidermal growth factor (*EGFR*) or *KRAS* genes. This is particularly useful when no biopsy is available for genetic analyses and, in this setting, the European Medicine Agency recommends *EGFR* testing in liquid biopsies to select patients for tyrosine kinase inhibitor (TKI) therapy (10). However, many standard techniques for mutation detection are not useful for ctDNA analyses due to an insufficient sensitivity. Since ctDNA often represents a small percentage of the total cfDNA, somatic mutations coming from the tumor can be present at allele fractions as low as 0.01%. Highly sensitive methodologies, or variations of preexisting methodologies, have been developed in order to detect low abundance mutations in cfDNA (6, 11).

Modified real-time PCR techniques have been widely used to identify genetic alterations in the cfDNA of cancer patients. They include amplification-refractory mutation system [ARMS (12),], Scorpion-ARMS (13), and peptide nucleic acid (PNA) or locked nucleic acid (LNA) mutant-enriched PCR (14–17). The diagnostic sensitivity of these techniques, when compared to tumor tissue, ranges from 43 to more than 90%, while the specificity is usually close to 100%; and the two commercially available methods to determine *EGFR* mutations in the cfDNA of cancer patients (Therascreen Plasma from Qiagen and COBAS Blood from Roche Diagnostics) are based on them. In our group, we have developed a quantitative PCR technique in the presence of PNA to detect *EGFR, KRAS*, and BRAF mutations in the cfDNA of advanced lung, colon, and cancer patients that achieves 75–80% sensitivity with 100% specificity (18, 19). Digital PCR, droplet digital PCR, and beads, emulsion, amplification, and magnetics (BEAMing) system constitute further refinements of the PCR-based techniques and have also been used to determine mutations in cfDNA (14, 20–26) (Table 2).

**Table 2:** **Summary of reports on detection of genetic alterations in liquid biopsy materials from advanced NSCLC patients**.

Technique	*n*	Type of sample	Alteration detected	Sensitivity (%)	Reference
ARMS	86	Circulating free DNA (cfDNA) (plasma)	Epidermal growth factor (EGFR)-sensitizing mutations	68	(27)
SARMS	42	cfDNA (serum)	EGFR-sensitizing mutations	75	(13)
SARMS	11	cfDNA (serum)	EGFR-sensitizing mutations	50	(13)
SARMS	21	cfDNA (plasma)	EGFR-sensitizing mutations	39	(28)
SARMS-based DxS EGFR mutation test kit	86	cfDNA (serum)	EGFR-sensitizing mutations	43	(15)
SARMS-based EGFR mutation detection kit	652	cfDNA (plasma)	EGFR-sensitizing mutations	66	(12)
Mass spectrometry-based genotyping	31	cfDNA (plasma)	EGFR-sensitizing mutations	39	(29)
Mutant-enriched PCR			EGFR-sensitizing mutations	33	
Mutant-enriched PCR	18	cfDNA (plasma)	EGFR-sensitizing mutations	100	(30)
Mutant-enriched PCR	111	cfDNA (plasma)	EGFR-sensitizing mutations	56	(31)
EGFR array, PNA-PCR	37	cfDNA (plasma)	EGFR-sensitizing mutations	100	(32)
Digital PCR	35	cfDNA (plasma)	EGFR-sensitizing mutations	92	(22)
Droplet digital PCR	46	cfDNA (plasma)	EGFR-sensitizing mutations	67	(33)
Droplet digital PCR	50	cfDNA (plasma)	EGFR mutations	76	(34)
Droplet digital PCR	25	cfDNA (plasma)	EGFR mutations	81	(35)
Cobas^®^ EGFR blood test	199	cfDNA (plasma)	EGFR-sensitizing mutations	61	(20)
Cobas^®^ EGFR blood test	38	cfDNA (plasma)	p.T790M (EGFR)	73	(36)
Cobas^®^ EGFR blood test	238	cfDNA (plasma)	EGFR mutations	76	(14)
DHPLC	230	cfDNA (plasma)	EGFR-sensitizing mutations	82	(37)
DHPLC	822	cfDNA (plasma)	EGFR-sensitizing mutations	77	(36)
BEAMing	44	cfDNA (plasma)	EGFR-sensitizing mutations	73	(24)
BEAMing^b^	915	cfDNA (plasma)	EGFR, KRAS, BRAF, PIK3CA mutations	83–99^c^	(23)
BEAMing	153	cfDNA (plasma)	EGFR-sensitizing mutations	82	(26)
			p.T790M	73	
Cobas^®^ EGFR blood test			EGFR-sensitizing mutations	73	
			p.T790M	64	
PNA-Q-PCR	97	cfDNA (serum/plasma)	EGFR sensitizing mutations	78	(18)
PNA/LNA-Q-PCR	35	cfDNA (serum)	EGFR, KRAS mutations	73	(17)
NGS (CAPP-Seq)	142	cfDNA (plasma)	EGFR mutations	81	(38)
NGS (Ion Torrent)^a^	107	cfDNA (plasma)	EGFR, HER2, KRAS, BRAF, PIK3CA mutations	58	(39)
NGS (deep sequencing)	288	cfDNA (plasma)	EGFR mutations	73	(40)
Melting curve PCR	8	Circulating tumor cells (CTCs)	EGFR mutations	100	(41)
NGS	37	CTCs	EGFR mutations	84	(42)
Mutant-enriched PCR	21	CTCs	p.T790M (EGFR)	57^c^	(43)
	25	cfDNA (plasma)		60^c^	
ISET + fluorescence *in situ* hybridization (FISH)	5	CTCs	ALK fusions	100	(44)
ISET + filter-adapted FISH	32	CTCs	ALK fusions	100	(45)
ISET + filter-adapted FISH	4	CTCs	ROS1 fusions	100	(46)
Antibody-independent CTC isolation + FISH	31	CTCs	ALK fusions	≥90^c^	(47)
NanoVelcro System + FISH	41	CTCs	ALK fusions	100	(48)
Retrotranscription PCR	77	cfRNA (plasma)	ALK fusions	22	(49)
		Platelets	ALK fusions	65	

*^a^Samples in the study include stages I–IIIA*.

*^b^Samples in the study include tumors other than NSCLC*.

*^c^Concordance value*.

Most modified PCR techniques are easy, comparatively unexpensive, and have a quick turnaround time (19), but have the disadvantage that can only detect mutations in a limited number of loci, usually within a single gene. Next-generation sequencing methodologies can overcome these limitations but, while tissue-based NGS genotyping is already well established, the application of NGS technologies to liquid biopsies is challenging and an ultra-deep sequencing approach is commonly used in order to improve sensitivity. In this approach, the gene panels are limited so that each read is sequenced thousands of times (39, 50, 51). However, this requirement of a high sensitivity may easily lead to false-positive results and requires a careful validation of the whole testing process. Examples of NGS protocols specifically developed for ctDNA analysis include TAm-Seq (tagged-amplicon deep sequencing), which combines site-specific primers with universal tails (52, 53); Safe-SeqS (Safe-Sequencing System) (54), and CAPP-seq (capture based sequencing), which relies on hybridization-based capture of target regions followed by amplification (38, 55) (Table 2).

The detection of mutations in cfDNA by modified PCR or NGS techniques is not only useful in lung cancer patients at presentation. It has also been successfully employed for patient monitoring, including early evaluation of response and relapse, which are associated with changes in the *EGFR* or *KRAS* mutational burden in cfDNA; and for early detection of acquired resistance to EGFR TKIs, associated in many patients with the emergence of the p.T790M mutation in blood (26, 56). In this respect, p.T790M testing in cfDNA has been recently recommended in patients eligible for osimertinib treatment, in order to avoid unnecessary rebiopsies (33, 36, 56).

## Circulating Tumor RNA

Similar to ctDNA, RNA derived from tumor cells (ctRNA) is present in the plasma of cancer patients and can be used for detection of the clinically relevant *ALK, ROS1*, and *RET* fusion genes and METΔ14 splicing variant. However, genetic analyses in cfRNA present specific challenges and have not been widely used. Unlike cfDNA, cfRNA degrades very quickly and needs to be purified rapidly after blood extraction. The alternative is adding a preservative such as Trizol and freezing the sample at −80°C, but this procedure is not easily accessible to many clinical sites. Despite these limitations, our group has a 5-year experience in detection of *EML4-ALK* fusion transcripts in plasma cfRNA by retrotranscription PCR (RT-PCR) (49) and, using improved processing and purification methods, we have demonstrated that the sensitivity of the technique can be significantly improved.

## Tumor-Educated Platelets

Platelets have been recently demonstrated to sequester tumor RNA by a microvesicle dependent mechanism, and the so-called TEPs (57, 58) can be used as a source of tumor RNA for genetic analysis. Platelets can be isolated from blood by simple centrifugation steps, and its RNA content easily purified and used for the detection of gene fusions and splicing variants. Using a RT-PCR approach, our group has detected *EML4-ALK* fusion transcripts in TEP RNA from advanced lung cancer patients with 65% sensitivity and 100% specificity (49). In addition, we have demonstrated that the disappearance of fusion transcripts in platelets correlates with response to crizotinib treatment. Regarding splicing variants, the clinical relevance of METΔ14 in lung cancer was only described in 2015 (59–61), and there are no reports in the literature about detection of METΔ14 transcripts in liquid biopsy. However, we have recently detected the alteration in the TEP RNA of a NSCLC patient positive in tumor tissue, who attained a partial response to crizotinib (unpublished data).

Platelet RNA can also be analyzed by multiplexing techniques, and a recent report has demonstrated the diagnostic potential of this approach. Using mRNA sequencing and surrogate TEP RNA profiles of 283 samples, 228 cancer patients of six different origins were discriminated from 55 healthy individuals with 96% accuracy. Tumors with specific genetic alterations, such as *KRAS* or *EGFR* mutations, were also distinguished and the location of the primary tumor identified with 71% accuracy (58).

## Exosomes

Exosomes are small vesicles present in blood and other body fluids (62–64). With a 30–100 nm diameter, they are constitutively released through exocytosis by many cells, including tumor cells, in physiological and pathological conditions. Exosomes contain lipids, proteins, mRNA, several types of non-coding RNAs, and double-stranded DNA; and their composition partly reflects that of the parental cells (65). In addition, being generated by the cell secretion pathway, all exosomes carry some common proteins independent of their origin, such as ALIX, CD63, or TSG-101 (66). Exosomes are generally isolated by sucrose gradient ultracentrifugation or immune-bead isolation techniques (such as magnetic activated cell sorting), and there are commercial kits available. Once isolated, exosomes are characterized by transmission electron microscopy, Western blot, FACS, or other methodologies (67).

Although being more difficult to purify than other materials, the lipid bilayer of exosomes makes their cargo particularly stable, theoretically allowing the identification of the tumor of origin, genetic alterations or resistances to treatments. In this respect, EML4-*ALK* fusion transcripts have been recently identified in the exosomal RNA of NSCLC patients (68). In addition, some studies indicate that micro RNA (miRNA) analysis of exosomes might be useful for the diagnosis of lung adenocarcinoma (69–71) and that particular miRNAs can offer prognostic information in advanced NSCLC. For example, downregulation of miRNA-373 and miRNA-512 has been associated with a poor prognosis (72), miR-208a and miR-1246 with resistance to radiotherapy (73, 74), and miR-221-3p and 222-3p with good response to osimertinib in *EGFR* mutated patients (75).

## Circulating Tumor Cells

Together with ctDNA, CTCs are the most widely investigated material in liquid biopsies of cancer patients. First observed in 1869 (76), they are cancer cells detached from the solid tumor mass that circulate in the blood and lymphatic system (77) as single cells or as aggregates, the so-called circulating tumor microemboli (78–80). In advanced NSCLC patients, CTCs are relatively rare, 1–10 per mL against a background of 10^6^–10^7^ peripheral blood mononuclear cells. This low abundance poses formidable challenges for the development of robust and sensitive enrichment protocols (81).

Some CTC capture methods are label dependent, based on specific epithelial cell surface markers, such as epithelial cell adhesion molecule (EpCAM) for positive selection or CD45 for negative depletion. One of such techniques, the CellSearch^®^ system (Veridex), has been approved by the FDA for monitoring some type of tumors (82–84), but not lung cancer. In advanced NSCLC, CellSearch^®^ has shown a limited detection efficiency, with CTCs detectable in only 20–40% of patients (85–87). Label-dependent methods do not select CTCs that have undergone epithelial to mesenchymal transition (88) or those with stem cell characteristics that have not started epithelial differentiation. Label-independent techniques, which are based on physical characteristics such as size, can overcome this limitation. Isolation by Size of Epithelial Tumor cells (ISET^®^, Rarecells), based on filtration and cytological characterization, has shown an increased sensitivity in NSCLC (89–92) with an 80% detection rate of CTCs in blood from 40 stage IIIA–IV patients compared with 23% using CellSearch^®^ (85). Another technology based on size, ScreenCell^®^, allows not only the detection but also the isolation of CTCs, which can be subjected to further morphological studies and used for genetic testing. Isolated CTCs can be cultured or injected into mice to generate xenografts (93–96) and CTC-derived tumor cells can thus be obtained in enough numbers for molecular and pharmacological profiling.

CTC counts have been extensively researched as a prognostic factor in NSCLC (97). In early-stage patients, the decrease or disappearance of CTCs after surgery has been reported to correlate with better clinical outcomes (98, 99), while its persistence was associated with shorter progression-free survival (PFS) (100). Regarding advanced NSCLC, some studies have reported that a higher CTC count at presentation correlates with advanced stage and shorter PFS and overall survival (85, 101). Also, the decrease or disappearance of CTCs after chemotherapy or targeted therapy has been consistently associated with better outcomes (102–104).

Finally, CTCs have also been investigated as a tool to identify clinically relevant genetic alterations in NSCLC (Table 2). Using NGS and modified PCR techniques, *EGFR*-sensitizing mutations and the p.T790M resistance mutation have been detected in the CTCs of *EGFR*-positive patients at presentation and after progression to TKI treatments, respectively (28, 41, 42, 105). The sensitivities reported range from 47 to 100%. However, unlike cfDNA, CTCs are not used for *EGFR* testing in the routine clinical practice. EML4-*ALK* fusions have been identified by fluorescence *in situ* hybridization (FISH) and immunochemistry in CTCs isolated using ISET (44) or other enrichment methodologies (47, 48). In some cases, filter-adapted FISH was employed, a methodology that has also been demonstrated to successfully identify ROS1 rearrangements in CTCs isolated by ISET (46).

## Author Contributions

All the authors contributed to the writing and critical revision of the review.

## Conflict of Interest Statement

The authors declare that this review was elaborated in the absence of any commercial or financial relationships that could be construed as a potential conflict of interest.
